# Thermally induced isomerization of linoleic acid and α‐linolenic acid in *Rosa roxburghii* Tratt seed oil

**DOI:** 10.1002/fsn3.2222

**Published:** 2021-05-04

**Authors:** Yongfu Li, Yuanshan Yu, Qiqi Luo, Yangbo He, Zhuxi Tian, Yueliang Zhao

**Affiliations:** ^1^ Integrated Rural Development Center Guizhou Academy of Agricultural Science Guiyang China; ^2^ Institute of Sericulture and Agricultural Products Processing Guangdong Academy of Agricultural Sciences Guagnzhou China; ^3^ College of Food Science and Technology Shanghai Ocean University Shanghai China

**Keywords:** heat treatment, isomerization, kinetics, *Rosa roxburghii* seed oil, *trans*‐fatty acids

## Abstract

*Rosa roxburghii* seed oil is obtained from the seeds left following pressing of the juice from *R. roxburghii* fruit. The total oil content of *R. roxburghii* seed was around 9.30%. The fatty acid profile of the oil was determined by gas chromatography (GC). Among the 11 fatty acids identified in the oil, seven were unsaturated fatty acids (UFAs) (92.6%); four were saturated fatty acids (SFAs) (7.17%). Then, the kinetics of formation of *trans*‐fatty acids was studied by GC. Heat treatment of *R. roxburghii* seed oil showed an increase in the relative percentage of linoleic acid and α‐linolenic acid isomers with increasing temperature and time. The formation of linoleic acid and α‐linolenic acid isomers followed a zero‐order reaction. The presence of O_2_ enhanced the isomerization of these UFAs. In addition, the rate constants and activation energies for the geometrical isomerization of UFAs in *R. roxburghii* seed oil were presented. Overall, *R. roxburghii* seed oil contains high UFA contents. Heating temperature and duration and the presence of O_2_ should be considered to reduce the formation of *trans*‐fatty acids during thermal treatment of *R. roxburghii* seed oil.

## Graphical abstract

*Rosa roxburghii* seed oil was of high quality in terms of its high amount of unsaturated fatty acids. Heat treatment of *R. roxburghii* seed oil showed an increase in the relative percentage of linoleic acid and linolenic acid isomers with increasing temperature and time. The formation of linoleic acid and linolenic acid isomers followed a zero order reaction.

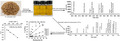

## 1 INTRODUCTION

*Rosa roxburghii* Tratt (*R. roxburghii*), belonging to the *Rosaceae* family, is a perennial deciduous shrub widely cultivated in Guizhou, Yunnan and Sichuan province, China (Fan et al., 1997; Wang et al., 2018). Its fruit juice has been consumed as a popular beverage and herbal tea in folk in China and claimed to possess functions of clearing summer heat, curing diarrhea, and tonifying spleen (Wu et al., 2020). *Rosa roxburghii* fruit have also been reported to exert antioxidant, antiatherogenic, antimutagenic, and radioprotective activities (He et al., 2016; Westhuizen et al., 2008; Xu et al., 2016). The health benefits have been attributed to the bioactive components including flavonoids, organic acids, triterpenes, and polysaccharides present in the *R. roxburghii* fruit (Liu et al., 2016; Xu et al., 2017; Zhu et al., 2019). The contents of total phenolics, total flavonoids, and ascorbic acid in *R. roxburghii* fruit were much higher than the selected fruits and vegetables, such as strawberry, blueberry, and lemon (Yang et al., 2020).

Generally, more than 50% of fruit typically becomes waste during *R. roxburghii* fruit juice production. *Rosa roxburghii* seed oil is obtained from the seeds left following pressing of the juice from *R. roxburghii* fruit. In regions with large *R. roxburghii* cultivation, the usage of *R. roxburghii* seed to make edible oil can generate extra benefits for juice production industries. Various methods including supercritical fluid extraction (SFE), Soxhlet extraction, and traditional solvent extraction with ultrasound have been conducted for *R. roxburghii* seed oil extraction. It was found that *R. roxburghii* seed oil was rich in unsaturated fatty acids, mainly linoleic acid (41.68%–74.34%), α‐linolenic acid (25.44%–41.11%), and oleic acid (12.74%–17.68%) (Shi et al., 2013; Wang & Chen, 1994; Zhang et al., 2007). Unsaturated fatty acids (UFAs) are valued for their beneficial health effects against coronary artery disease, high cholesterol levels, cardiovascular diseases, and cancer (Mericli et al., 2017; Oomah et al., 2000; Vos & Cunnane, 2003). Thus, *R. roxburghii* seed oil was of high quality in terms of its high UFAs contents.

It is well known that heat treatment such as frying can cause profound changes in the chemical composition of oils (Cui et al., 2017; Li et al., 2012). UFAs can be isomerize into more stable *trans*‐fatty acids (TFAs) by thermal treatment (Christy & Arachchi, 2016; Christy et al., 2009), which is shown to accumulate in liver, heart, and many other organs (Ganguly et al., 2016), causing increased risks of coronary heart disease and type‐2 diabetes (Ascherio et al., 1999; Papantoniou et al., 2010). Kinetics of geometrical isomerization of UFAs has been reported by several research groups. The geometrical isomerization of linoleic and α‐linolenic acid in heated soybean oil followed a first‐order reaction (Gerčar & Šmidovnik, 2002). Heat treatment of sunflower oil showed an increase in the relative percentage of *trans*‐linoleic acid with increasing temperature and time and the formation of *trans*‐linoleic acid isomers followed zero‐order kinetics (Mateos et al., 2010). Studies on the thermally induced isomerization kinetics of 9c,12c linoleic acid in triacylglycerol showed that the consumption of trilinolein followed a second‐order reaction and the formation reactions of *cis*,*trans, trans*,*cis* and *trans*,*trans* isomers followed zero‐order kinetics and dependent on both heating temperature and total heating time (Guo et al., 2016).

*Rosa roxburghii* seed oil contains high amount of unsaturated fatty acids; however, till now, no scientific study on the kinetics of isomerization of linoleic and α‐linolenic acids during heat treatment has been published. The objective of the present study is to (a) determine the composition of fatty acids in *R. roxburghii* seed oil and (b) investigate the kinetics of geometrical isomerization of unsaturated fatty acids in *R. roxburghii* seed oil in the presence of air (O_2_) or in nitrogen (N_2_) atmosphere during heat treatment using GC. The kinetic parameters determined for the thermally induced *cis*‐*trans* isomerization may provide insight into methods for controlling isomerization reactions and manipulating isomeric yield ratios.

## 2 MATERIALS AND METHODS

### 2.1 Materials

Mixture of 37 fatty acid methyl esters (FAME), linoleic acid methyl ester (LAME) mixture (9c,12c; 9c,12t; 9t,12c; 9t,12t), α‐linolenic acid methyl ester mixture (9t,12t,15t; 9t,12t,15c; 9t,12c,15t; 9c,12t,15t; 9c,12c,15t; 9t,12c,15c; 9c,12t,15c; 9c,12c,15c), and potassium hydroxide (KOH) were purchased from Sigma‐Aldrich. Isooctane (chromatographic grade) was obtained from Aladdin CO. Hexane and methanol of HPLC grade were purchased from Merck KGaA.

### 2.2 Extraction of *R. roxburghii* seed oil

*Rosa roxburghii* seeds harvested in autumn 2019 were provided by Guizhou Lvyuan Food Co. Ltd. One hundred gram of pulverized *R. roxburghii* seeds was ultrasonic extracted in hexane with a solid–liquid ratio of 1:3. The extraction temperature was 45°C, ultrasonic power was 200 W, and extraction time was 30 min. The extraction procedure was repeated for three times. After that, the extraction solutions were combined and evaporated to a constant weight using a rotary evaporator. The organic solvent residue was further removed via nitrogen stream. The extracted oil was then decolorized with kaolin prior to GC analysis and heat treatment.

### 2.3 Thermal processing

Each 4 ml microglass ampoule bottle was filled with 100 mg of *R. roxburghii* seed oil. The ampoules with air or nitrogen (N_2_, 5 ml/min, 5 min) in the head space were sealed with a propane‐oxygen flame. The sealed oil samples were then heated in a silicone oil bath at 180, 200, 220, 230, or 240°C (±2°C) for regular time intervals. After heat treatment, the samples were cooled to room temperature before further analysis.

### 2.4 Preparation of fatty acid methyl esters

Fatty acid methyl esters were prepared according to a previous study (Guo et al., 2015). Briefly, 100 mg of the *R. roxburghii* seed oil sample and 0.05 ml of 2 mol/L methanolic KOH were mixed thoroughly using a vortex. Then, the vials were centrifuged at 4,000 × g for 10 min. The supernatants were dried with anhydrous magnesium sulfate. After that, 20 μl of the dried supernatant was diluted to 1 ml with isooctane. One microliter of the supernatant was injected into a gas chromatograph (GC) for fatty acid analysis.

### 2.5 GC‐FID analysis

GC‐FID was applied to quantify fatty acid in soybean oil during heat treatment (Guo et al., 2015; Liu et al., 2021). GC (TRACE 3000, Thermo Fisher Inc.) was equipped with an ionic liquid SLB‐IL111 column (100 m × 0.25 mm × 0.2 mm), and a flame ionization detector (FID, Thermo Fisher Inc.). Helium (99.999%) was used as the carrier gas with a flow rate of 1.0 ml/min. Column temperature program was as follows: 60°C (5 min), 60–160°C at 25°C/min, 160°C (5 min), 160–225°C at 1.5°C/min, and 225°C (15 min). The temperature for the injector was 230°C. The injection volume was 1 μl with a split ratio of 1:10. Fatty acids were identified by comparison of retention time with those of commercial standards. The quantitation of the fatty acids was performed using external standard method. The content of fatty acid was expressed as g/100 g of *R. roxburghii* seed oil.

### 2.6 Statistical analysis

Results were expressed as mean ± standard deviation of at least three independent experiments. Statistical analysis was carried out using ANOVA by Prism 5.0, GraphPad Software. The significance of differences was calculated using the Student's paired *t* test. *p*‐value < .05 was considered statistically significant.

## 3 RESULTS AND DISCUSSION

### 3.1 Fatty acid composition

The oil in *R. roxburghii* seed was ultrasonic extracted with hexane. The total oil content of *R. roxburghii* seed was around 9.30%, which is lower compared with seeds of fruit such as chakeberry (19.3%) and blackcurrant (22.0%), and similar to those of grape seed (around 10%) and rosehip seed (8.20%) (Fernandes et al., 2013; Matthaus & Özcan, 2011).

**FIGURE 1:**
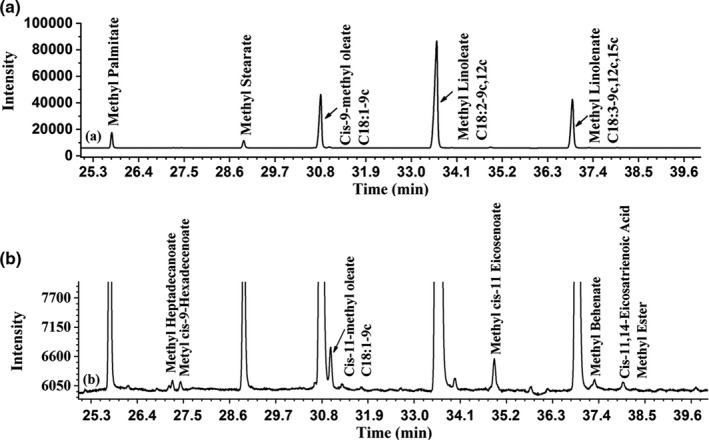
Gas chromatography (GC) spectrum of FAME composition of *R. roxburghii* seed oil

The fatty acid profile of *R. roxburghii* seed oil was determined by GC (Figure 1). Fatty acid compositions of the *R. roxburghii* seed were illustrated in Table 1. Among the 11 fatty acids identified in *R. roxburghii* seed oil, 7 were unsaturated fatty acids (92.6%); four were saturated fatty acids (7.17%). The most abundant fatty acids were three unsaturated fatty acids including linoleic acid (52.0%), α‐linolenic acid (20.1%), and oleic acid (20.1%). These values were close to those obtained by previous study (linoleic acid: 41.68%–74.34%; α‐linolenic acid: 25.44%–41.11%; oleic acid: 12.74%–17.68%) (Shi et al., 2013; Wang & Chen, 1994; Zhang et al., 2007). Moreover, the unsaturated fatty acid content of *R. roxburghii* seed oil was more than rapeseed oil, peanut oil, soybean oil, and especially pig oil and safflower seed oil (Ma et al., 2011). In addition, the unsaturated fatty acid content of *R. roxburghii* seed oil was similar with olive oil which was characterized by high unsaturated fatty acid content (around 90%). *Rosa roxburghii* seed oil contains higher PUFA (72.12% vs. 6.0%–15.9%) but lower MUFA (20.47% vs. 64.4%–81.0%) compared with olive oil (Maggio et al., 2009). It is worth mentioning that the high amount of unsaturated fatty acids especially linoleic and α‐linolenic acid makes *R. roxburghii* seed oil susceptible to oxidation. Yet, these unsaturated fatty acids were of high nutritional values and have beneficial physiological effects against coronary heart disease and cancer (Oomah et al., 2000).

**TABLE 1:** Fatty acid composition of *R. roxburghii* seed oil

Fatty acids	Concentration (g/100 g)	Concentration (%)
Palmitic acid (C16:0)	2.489 ± 0.028	4.47 ± 0.05
Margaric acid (C17:0)	0.043 ± 0.0003	0.0770 ± 0.001
*Cis*‐9‐heptadecenoic acid (C17:1‐9c)	0.039 ± 0.0018	0.0710 ± 0.003
Stearic acid (C18:0)	1.412 ± 0.038	2.54 ± 0.07
oleic acid (C18:1‐9c)	11.168 ± 0.031	20.1 ± 0.06
Asclepic acid (C18:1‐11c)	0.196 ± 0.0008	0.352 ± 0.001
Linoleic acid (C18:2‐9c,12c)	28.941 ± 0.099	52.0 ± 0.18
α‐Linolenic acid (C18:3‐9c,12c,15c)	11.184 ± 0.052	20.1 ± 0.09
Docosanoic acid (C22:0)	0.048 ± 0.0007	0.0890 ± 0.001
Gadoleic acid (C20:1‐11c)	0.135 ± 0.0015	0.241 ± 0.003
*Cis*‐11, 14‐eicosadienoic acid (C20:2‐11c,14c)	0.049 ± 0.0011	0.0920 ± 0.002
Total SFA	3.992 ± 0.067	7.17 ± 0.12
Total MUFA	11.403 ± 0.034	20.5 ± 0.06
Total PUFA	40.174 ± 0.152	72.1 ± 0.27
Total fatty acids	55.704	

### 3.2 Analysis of *trans*‐fatty acids in heated *R. roxburghii* seed oil by GC

**FIGURE 2:**
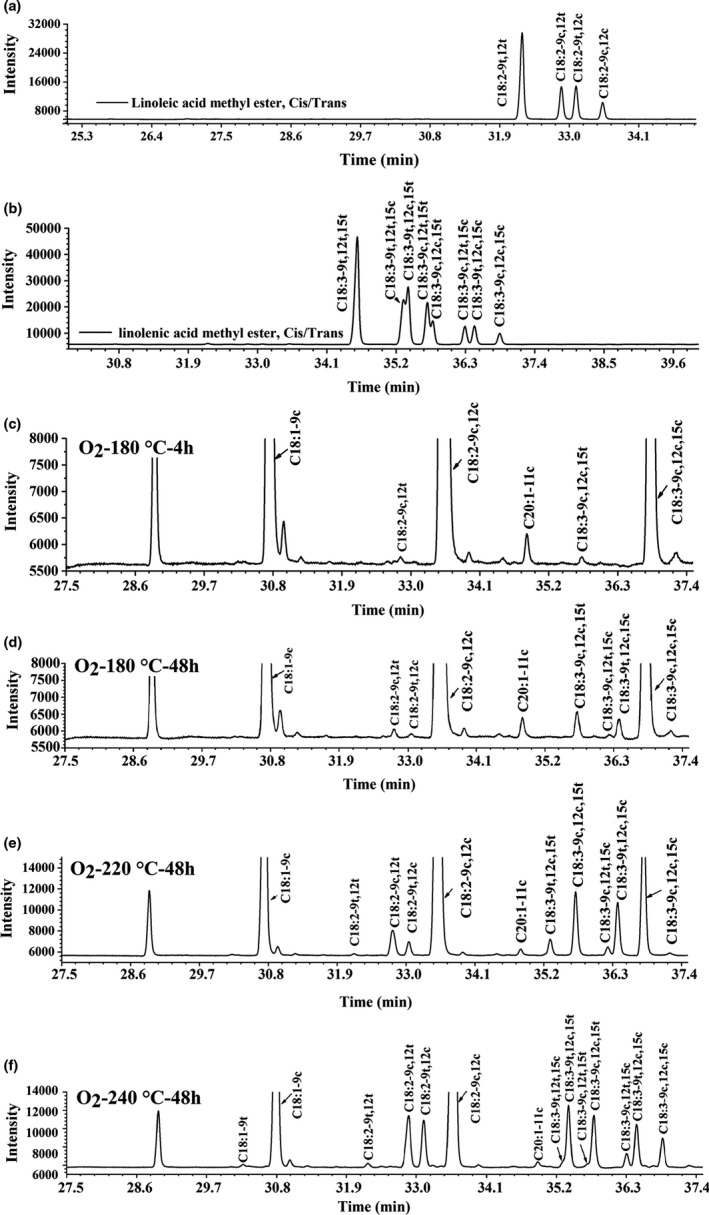
GC spectrum of (a) linoleic acid methyl ester, *cis‐trans* isomers; (b) α‐linolenic acid methyl ester, *cis‐trans* isomers; and (c–f) fatty acids isomers generated in *R. roxburghii* seed oil during heating at different temperature and time in the presence of air (O_2_)

The gas chromatography spectrum of linoleic acid isomers or α‐linolenic acid isomers was presented in Figure 2a,b, respectively. The peaks ofα‐linoleic acid isomers were well separated, and easily distinguished (Figure 2a). For the α‐linolenic acid isomers, C18:3‐9t,12t,15t, C18:3‐9t,12c,15c, C18:3‐9c,12t,15c, and C18:3‐9c,12c,15c were well separated. However, the retention times of C18:3‐9t,12t,15c and C18:3‐9t,12c,15t were close to each other. There are some overlaps between the peaks of C18:3‐9c,12t,15t and C18:3‐9c,12c,15t (Figure 2b). Quantitative analysis of overlapping peaks was conducted by determining the area of each half peak.

The isomerization of double bonds requires rotational energy (Tsuzuki, 2010). A range of *trans*‐linoleic and *trans*‐linolenic acids were observed in thermal‐treated *R. roxburghii* seed oil in the presence of air. The amount of each *trans*‐fatty acid (TFA) increased with increasing temperature and time (Figure 2). The *cis‐trans* isomerization of C18:2‐9c,12c induced the formation of C18:2‐9c,12t, whereas the *trans* isomers of C18:3‐9c,12c,15c was C18:3‐9c,12c,15t in *R. roxburghii* seed oil heated at a relative low temperature (180°C) for 4 hr (Figure 2c). The content of C18:3‐9c,12c,15t was higher than C18:2‐9c,12t. These results suggested that unsaturated fatty acids with more double bond are more likely undergo thermal isomerization and double bond closest to the methyl end is more likely to be thermally isomerized. Moreover, isomers with two *trans* double bonds such as C18:2‐9t,12t, C18:3‐9t,12c,15t, C18:3‐9t,12t,15c, and C18:3‐9t,12c,15t were formed in *R. roxburghii* seed oil heated at 220 or 240°C for 48 hr but not in *R. roxburghii* seed oil heated at 180°C for 4 or 48 hr, suggesting that the formation of isomers with two *trans* double bonds requires more energy than the formation of isomers with one *trans* double bond (Li et al., 2012).

### 3.3 Kinetics for the formation of C18:2‐9c,12t, C18:2‐9t,12c, and C18:2‐9t,12t isomers

**FIGURE 3:**
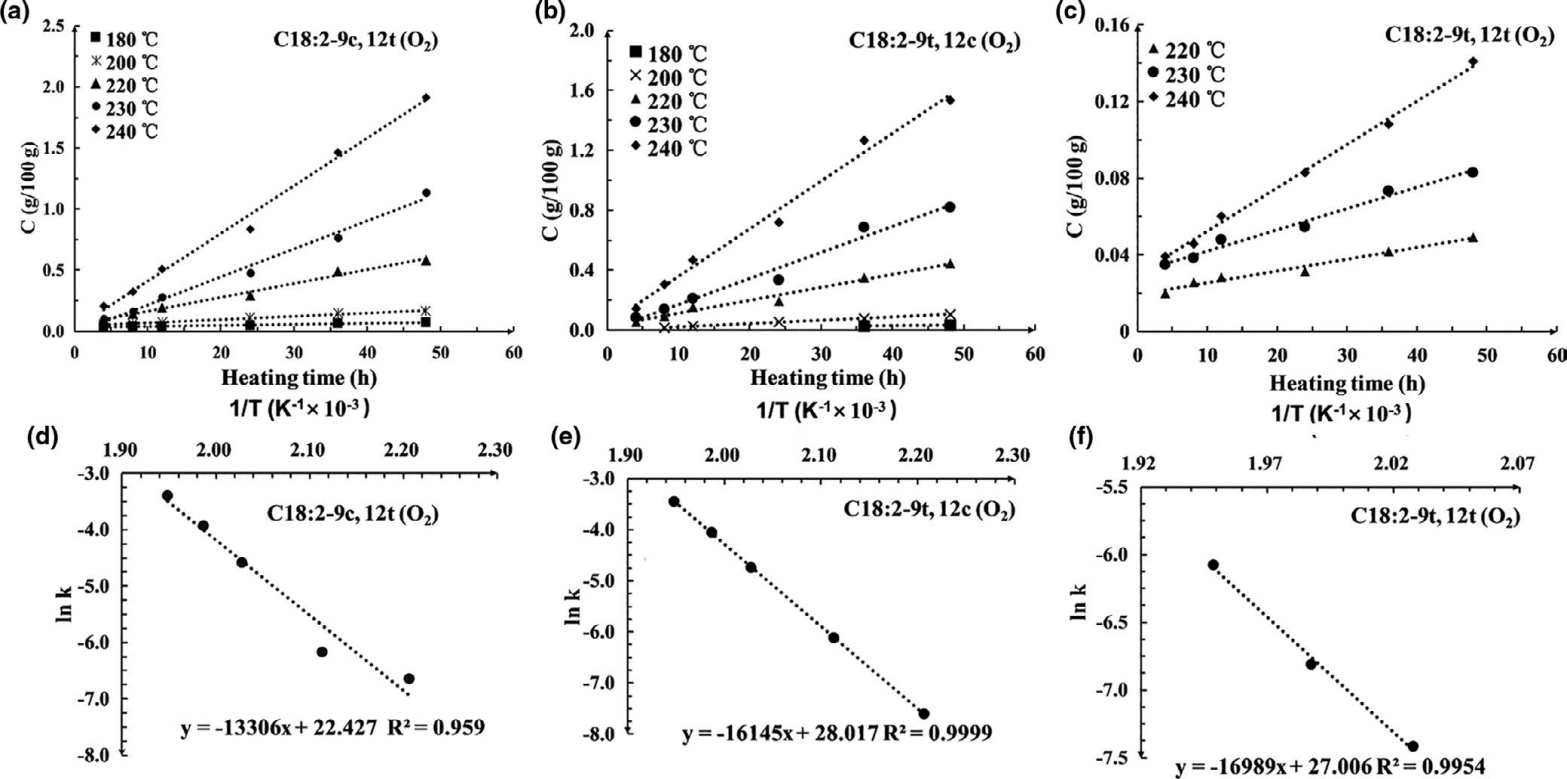
Plots showing the correlation between [C] and time for the (a) C18:2‐9c,12t, (b) C18:2‐9c,12t, and (c) C18:2‐9c,12t isomers in the presence of N_2_, and the relationship between lnk and 1/T for the (d) C18:2‐9c,12t and (e) C18:2‐9c,12t, and (f) C18:2‐9c,12t isomers in the presence of N_2_ at different temperatures

**FIGURE 4:**
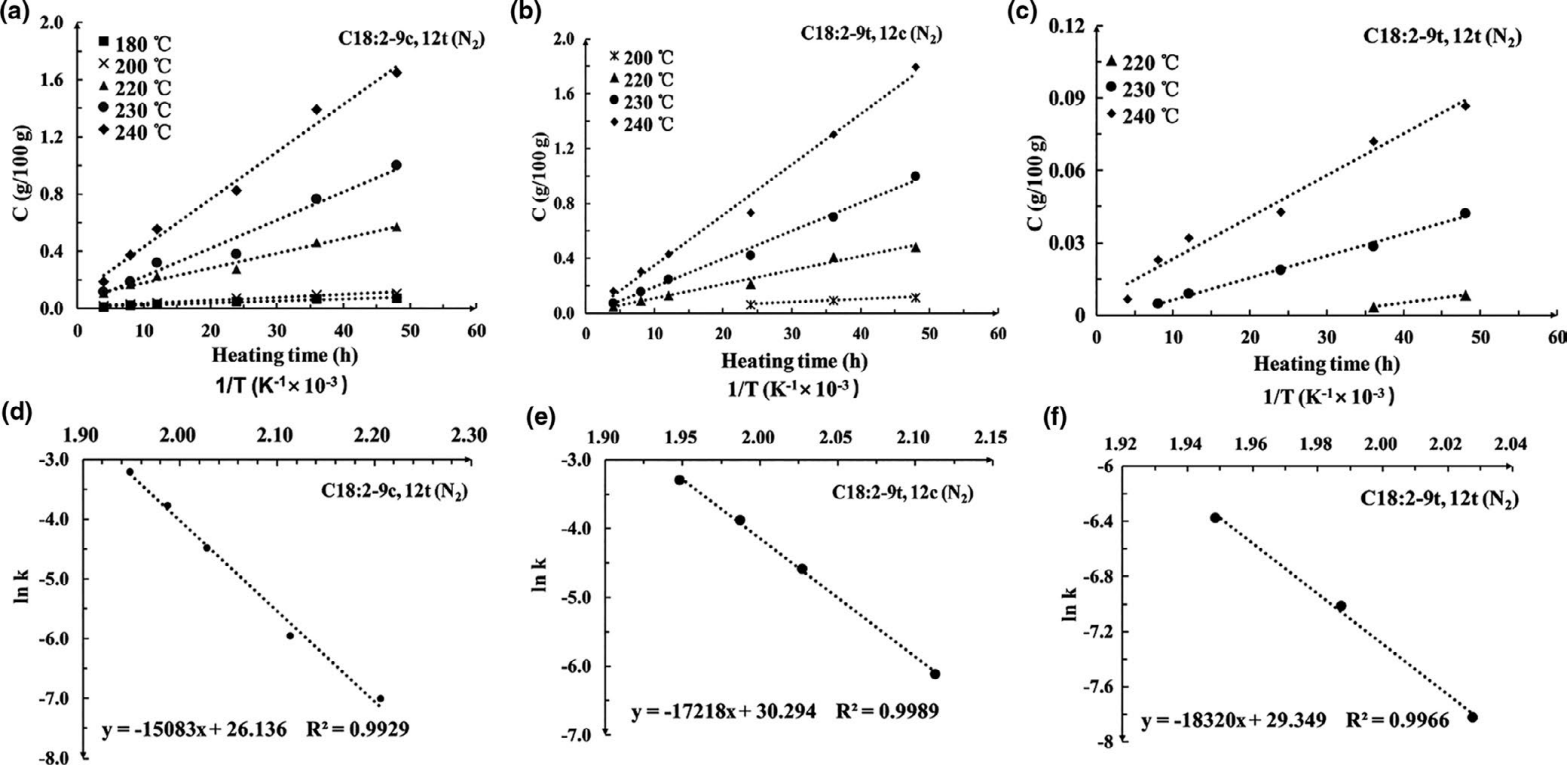
Plots showing the correlation between [C] and time for the (a) C18:2‐9c,12t, (b) C18:2‐9c,12t, and (c) C18:2‐9c,12t isomers in the presence of O_2_, and the relationship between lnk and 1/T for the (d) C18:2‐9c,12t and (e) C18:2‐9c,12t, and (f) C18:2‐9c,12t isomers in the presence of O_2_ at different temperatures

The concentration evolution plots for C18:2‐9c,12t, C18:2‐9t,12c, and C18:2‐9t,12t during heating of *R. roxburghii* seed oil were shown in Figure 3a–c (oil heated in the presence of air) and Figure 4a–c (oil heated in N_2_ atmosphere). The relationships show that the formation of C18:2‐9c,12t, C18:2‐9t,12c, and C18:2‐9t,12t followed zero‐order kinetics in all tested heating temperature in the presence of air or in the presence of N_2_, which is in agreement with the reports of Guo (Guo et al., 2016). The amount of isomer formation can be calculated using Equation 1. Arrhenius plots of lnk with respect to 1/T were shown in Figures 3d–f and 4d–f, which demonstrated excellent linear correlation. Thus, the activation energy (*E*_a_) for the isomerization reaction can be relatively precisely determined by Equation 2.

(1) $$C_{\text{isomer}}=k\cdot t$$

(2) $$\ln\;k=‐\frac{E_{a}}{R}\cdot \frac{1}{T}+c$$

The kinetic parameters for the formation of C18:2‐9c,12t, C18:2‐9t,12c, and C18:2‐9t,12t isomers were presented in Table 2. The *k* values for all isomers were increased with increasing temperature, suggesting that increasing heating temperature enhanced the isomerization reaction. The *k* values of isomers formed in the presence of air were higher than that in the presence N_2_, indicating that O_2_ assisted the isomerization of linoleic acid. The *E*_a_ for the formation of the 9c,12t isomer was much lower than that of the 9t,12c isomer. 9t,12t isomer has higher *E*_a_ than 9t,12c isomer in the same conditions, further confirming that double bond closest to the methyl end is much easier to be thermally isomerized and the formation of isomers with two *trans* double bonds requires more energy than the formation of isomers with one *trans* double bond (Li et al., 2012). In agreement with our findings, C18:2‐9t,12t isomer was reported to be formed from C18:2‐9c,12t and C18:2‐9t,12c in a previous study (Jiang et al., 2017). In addition, the *E*_a_ values for the formation of the isomers in presence of N_2_ atmosphere were much higher than that in the presence of air in the same heating temperature, further confirming that O_2_ enhanced the isomerization of linoleic acid.

**TABLE 2:** Kinetic parameters for the formation of linoleic acid isomers (9c,12t), (9t,12c), and (9t,12t)

	Reaction	9c,12t		9t,12c		9t,12t	
	T (°C)	*k* (g/100 g)^−1^ hr^−1^	*E*_a_ (kJ/mol)	*k* (g/100 g)^−1^ hr^−1^	*E*_a_ (kJ/mol)	*k* (g/100 g)^−1^ hr^−1^	*E*_a_ (kJ/mol)
O_2_	180	1.30 × 10^–3^	110.63	5.00 × 10^–4^	134.23	—	141.25
	200	2.10 × 10^–3^		2.20 × 10^–3^		—	
	220	1.03 × 10^–2^		8.70 × 10^–3^		6.00 × 10^–4^	
	230	1.98 × 10^–2^		1.75 × 10^–2^		1.10 × 10^–3^	
	240	3.33 × 10^–2^		3.18 × 10^–2^		2.30 × 10^–3^	
N_2_	180	9.00 × 10^–4^	125.40	—	143.15	—	152.31
	200	2.60 × 10^–3^		2.20 × 10^–3^		—	
	220	1.13 × 10^–2^		1.01 × 10^–2^		4.00 × 10^–4^	
	230	2.29 × 10^–2^		2.05 × 10^–2^		9.00 × 10^–4^	
	240	4.03 × 10^–2^		3.67 × 10^–2^		1.70 × 10^–3^	

### 3.4 Kinetics for the formation of C18:3‐9c,12c,15t, C18:3‐9t,12c,15c, C18:3‐9c,12t,15c, and C18:3‐9c,12t,15t isomers

**FIGURE 5:**
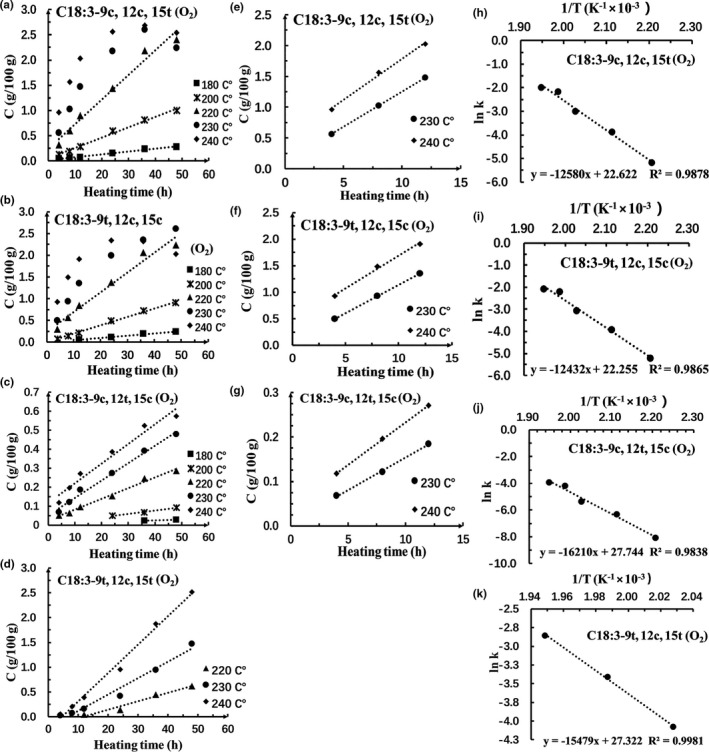
Plots showing the correlation between [C] and time for the (a–g) C18:3‐9c,12c,15t, C18:3‐9t,12c,15c, C18:3‐9c,12t,15c, and C18:3‐9t,12c,15t isomers in the presence of O_2_, and the relationship between lnk and 1/T for the (h–k) C18:3‐9c,12c,15t, C18:3‐9t,12c,15c, C18:3‐9c,12t,15c, and C18:3‐9c,12t,15t isomers in the presence of O_2_ (h–k)at different temperatures

**FIGURE 6:**
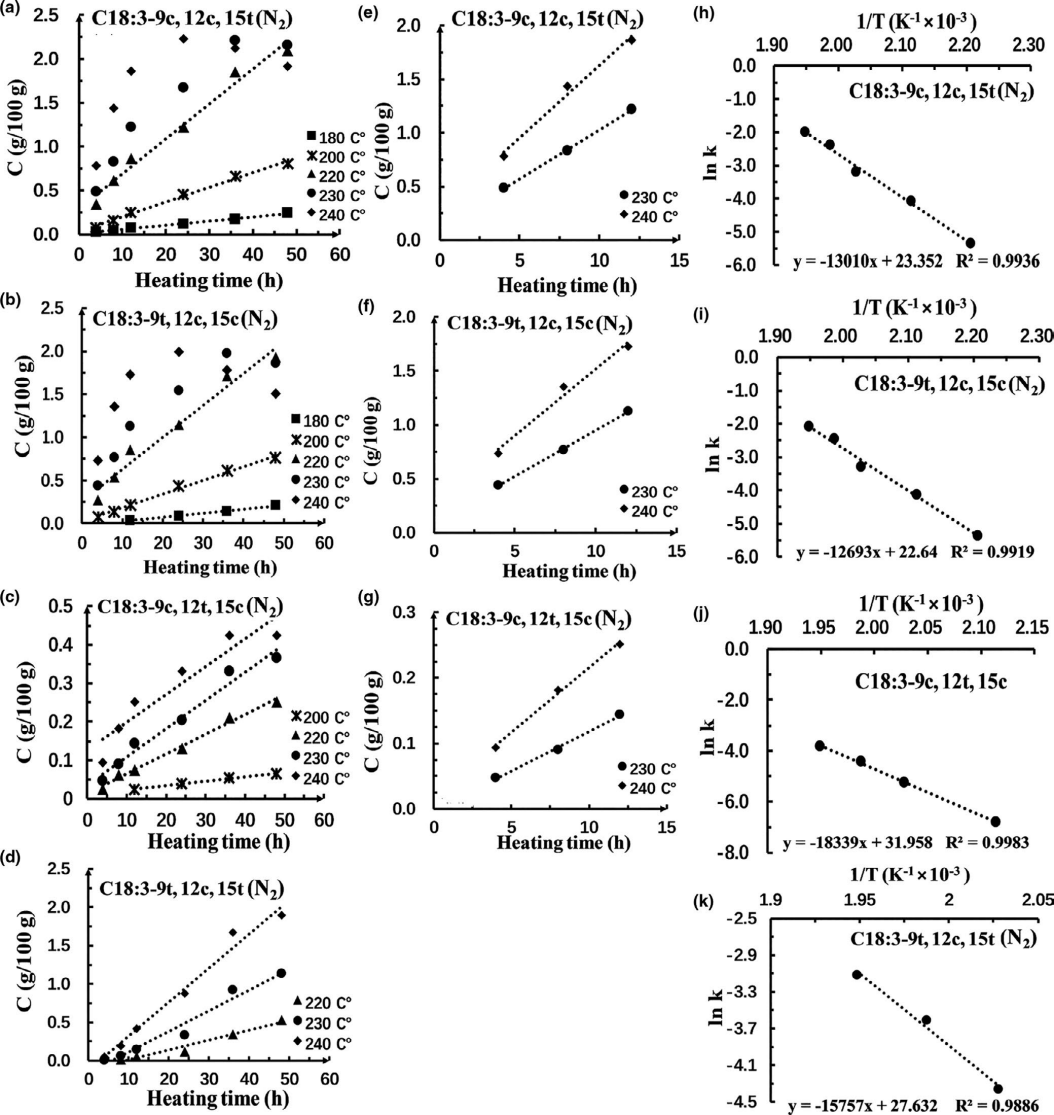
Plots showing the correlation between [C] and time for the (a–g) C18:3‐9c,12c,15t, C18:3‐9t,12c,15c, C18:3‐9c,12t,15c, and C18:3‐9t,12c,15t isomers in the presence of N_2_, and the relationship between lnk and 1/T for the (h–k) C18:3‐9c,12c,15t, C18:3‐9t,12c,15c, C18:3‐9c,12t,15c, and C18:3‐9c,12t,15t isomers in the presence of N_2_ (h–k)at different temperatures

We also studied the kinetics of geometrical isomerization of α‐linolenic acid in *R. roxburghii* seed oil in the presence of air or in the presence of N_2_ during heat treatment by GC. The formation of *trans* isomers of α‐linolenic acid such as C18:3‐9t,12c,15c and C18:3‐9c,12c,15t increased linearly with heating time at the temperature of ≤220°C (Figures 5a,b and 6a,b). However, there is not a linear correlation between the formation of these *trans* isomers and heating time when the oil was heating above 220°C (Figures 5a–c and 6a–c), which may be caused by the decomposition, oxidation, polymerization, and hydrolysis of isomers at extreme high temperature (Gerčar & Šmidovnik, 2002). It should be noted that when the heating time was <24 hr, there is an excellent linear correlation between the formation of C18:3‐9t,12c,15c, C18:3‐9c,12c,15t, and C18:3‐9c,12t,15c and heating time (Figures 5e–g and 6e–g). Thus, the formation kinetics of C18:3‐9t,12c,15c, C18:3‐9c,12c,15t, and C18:3‐9c,12t,15c within 24 hr of heating were studied.

The kinetic parameters for the formation of C18:3‐9t,12c,15c, C18:3‐9c,12c,15t, and C18:3‐9c,12t,15c isomers were presented in Table 3. Similar to the finding on isomerization of linoleic acid, the *k* values for c18:3‐9t,12c,15c, C18:3‐9c,12c,15t, and C18:3‐9c,12t,15c isomers were increased with increasing temperature and the *k* values of isomers formed in the presence of air were higher than that in the presence N_2_. These results further confirmed that increasing heating temperature and the presence of O_2_ could speed up the isomerization of unsaturated fatty acid. *E*_a_ value for the formation of α‐linolenic acid isomers was determined from the parameters of *k* and 1/T (Figures 5h–k and 6h–k). The *E*_a_ value for the formation of C18:3‐9c,12c,15t and C18:3‐9t,12c,15c was almost the same (Table 3) in samples in the presence of air or in the presence of N_2_. The *E*_a_ values for the formation of the isomers in presence of N_2_ atmosphere were much higher than that in the presence of air in the same heating temperature, suggesting that O_2_ promoted the isomerization of linoleic acid. The *E*_a_ value for the formation of C18:3‐9c,12t,15c was much higher than the *E*_a_ value for the formation of C18:3‐9c,12c,15t and 9t,12c,15c in samples in the presence of air or in the presence of N_2_, indicating that double bound at C12 position was the most difficult one to be thermally isomerized forming single trans‐fatty acids. In addition, compared Tables 2 and 3 we can find that geometrical isomerization of α‐linolenic acid requires less energy than linoleic acid, which could be one explanation for the formation of *trans*‐linolenic acid at a relative lower temperature than *trans*‐linoleic acid as mentioned in Section 3.2.

**TABLE 3:** Kinetic parameters for the formation of α‐linolenic acid isomers (9c,12c,15t), (9t,12c,15c), (9c,12t,15c), and (9c,12t,15t)

	Reaction	9c,12c,15t		9t,12c,15c		9c,12t,15c		9c,12t,15t	
	T (°C)	*k* (g/100 g)^−1^ hr^−1^	*E*_a_ (kJ/mol)	*k* (g/100 g)^−1^ hr^−1^	*E*_a_ (kJ/mol)	*k* (g/100 g)^−1^ hr^−1^	*E*_a_ (kJ/mol)	*k* (g/100 g)^−1^ hr^−1^	*E*_a_ (kJ/mol)
O_2_	180	5.60 × 10^–3^	104.59	5.40 × 10^–3^	103.34	3.00 × 10^–4^	134.77	—	128.69
	200	2.06 × 10^–2^		1.94 × 10^–2^		1.80 × 10^–3^		2.00 × 10^–3^	
	220	4.88 × 10^–2^		4.56 × 10^–2^		5.60 × 10^–3^		1.69 × 10^–2^	
	230	1.14 × 10^–1^		1.08 × 10^–1^		1.47 × 10^–2^		3.30 × 10^–2^	
	240	1.34 × 10^–1^		1.23 × 10^–1^		1.92 × 10^–2^		5.74 × 10^–2^	
N_2_	180	4.70 × 10^–3^	108.16	4.70 × 10^–3^	108.01	—	152.52	—	131.01
	200	1.68 × 10^–2^		1.59 × 10^–2^		1.10 × 10^–3^		2.10 × 10^–3^	
	220	4.06 × 10^–2^		3.71 × 10^–2^		5.30 × 10^–3^		1.28 × 10^–2^	
	230	9.15 × 10^–2^		8.55 × 10^–2^		7.30 × 10^–3^		2.71 × 10^–2^	
	240	1.35 × 10^–1^		1.24 × 10^–1^		8.80 × 10^–3^		4.44 × 10^–2^	

## 4 CONCLUSION

*Rosa roxburghii* seed, which is generally a waste material, is a good natural resource of high‐quality edible oil due to the fact that *R. roxburghii* seed oil contains high amount of UFAs (92.83%). Heat treatment of *R. roxburghii* seed oil showed an increase in the contents of linoleic acid and α‐linolenic acid isomers with increasing temperature and time. The formation of linoleic acid and α‐linolenic acid isomers followed a zero‐order reaction. The presence of O_2_ enhanced the isomerization of these UFAs. The thermal isomerization degree of UFAs depends on the number and position of the double bonds. Overall, *R. roxburghii* seed oil could be used as high‐quality edible oil because of its high UFAs contents. Heating temperature and duration and the presence of O_2_ should be considered to reduce the formation of *trans*‐fatty acids during thermal treatment *R. roxburghii* seed oil.

## CONFLICT OF INTEREST

The authors confirm that they have no conflicts of interest with respect to the work described in this manuscript.
